# Perceived Social Support and Depression, Anxiety and Stress in Pregnant Women Diagnosed With Foetal Anomaly

**DOI:** 10.1111/jan.16587

**Published:** 2024-11-04

**Authors:** Meltem Mecdi Kaydırak, Elif Balkan, Nilgul Bacak, Filiz Kızoglu

**Affiliations:** ^1^ Florence Nightingale Faculty of Nursing, Department of Women Health and Diseases Nursing Istanbul University‐Cerrahpasa Istanbul Turkey; ^2^ Faculty of Health Sciences, Department of Obstetrics and Gynecology Nursing Yalova University Yalova Turkey; ^3^ Department of Nursing Istanbul Health and Technology Unıversıty Istanbul Turkey; ^4^ Istanbul Faculty of Medıcıne Istanbul Unıversıty Istanbul Turkey

**Keywords:** anxiety, depression, maternity nursing, pregnancy, stress

## Abstract

**Aim:**

To examine the relationship between perceived social support level and depression, anxiety and stress in pregnant women diagnosed with foetal anomaly.

**Design/Methods:**

This descriptive and correlational study was conducted in an advanced prenatal evaluation unit of a university hospital between December 2021 and May 2022. The study data collected from 131 pregnant women through a personal information form, depression, anxiety and stress scale (DASS‐42) and multidimensional scale of perceived social support (MSPSS).

**Results:**

Most of the pregnant women were in the second trimester of pregnancy, and more than half had been advised by a healthcare professional to terminate their pregnancy. Overall, the pregnant women reported moderate levels of social support, while their depression, anxiety and stress levels varied. There was a weak negative correlation between perceived social support from family, friends and multidimensional sources with stress, but the effect rate was low.

**Conclusion:**

Most pregnant women diagnosed with foetal anomaly have normal levels of depression, stress and anxiety. There is a weak negative correlation between perceived social support and stress, with family and friend support affecting stress levels at a low rate. Professional support should be provided, and both the woman's mental health and social support mechanisms must be evaluated.

**Impact:**

This study highlights the importance of social support in managing stress among pregnant women with foetal anomalies. While most women had normal levels of depression, anxiety and stress, increased social support from family and friends was shown to reduce stress. The findings underscore the need for healthcare professionals to assess and strengthen mental health and social support systems in this vulnerable population, informing interventions to improve psychosocial outcomes.

**Reporting Method:**

This descriptive and correlational study adhered to the CONSORT guidelines for reporting non‐randomised trials.

**Patient or Public Contribution:**

No patient or public contribution.

## 1 Introduction

Structural, functional, behavioural and metabolic abnormalities that occur before birth and can be detected before, during or after birth or even in infancy are called congenital anomalies (World Health Organization 2016). A congenital anomaly is typically acknowledged as a defect that may cause the baby to die right after birth, or a condition that might require treatment prior or after birth to improve health and support survival (National Health Service 2015). Congenital abnormalities are considered a major threat to public health as they may result in abortion, foetal death, infant mortality and disabilities in childhood (WHO 2016; Kancherla, Oakley, and Brent 2014). Although antenatal screening has significantly increased the detection of major fatal abnormalities during pregnancy (Irving et al. 2011), the incidence of foetal anomaly varies in different regions of the world, but most neonatal deaths arise from foetal anomalies (Leung et al. 2004; Altın 2009). Approximately 3% of pregnancies worldwide are diagnosed with a major congenital abnormality (Coleman 2015). Of births, 6% result in foetal anomaly, with 60% of these in underdeveloped countries (Mandiracioglu 2011). In Ireland, a quarter of stillbirths and more than half of neonatal deaths in 2017 had a major congenital anomaly as the main cause of death (O'Farrell et al. 2019). In the United States, one in every 33 babies born with foetal anomalies (Center for Disease Control 2022).

## 2 Background

The disclosure of a diagnosis of a life‐threatening congenital malformation of the foetus during pregnancy usually comes as a great shock to the expectant parents (Benute et al. 2012; Fleming et al. 2016). Diagnosis of foetal anomaly cause sadness, depression, anxiety, grief and post‐traumatic stress for the pregnant woman dreaming of a healthy pregnancy and baby (Grenn and Strothom 1999; Karabulutlu and Yavuz 2021). Parents dealing with fatal foetal anomalies are increased risk for perinatal depression, traumatic stress and anxiety (Dempsey et al. 2021; Oftedal et al. 2022). In foetal anomalies incompatible with life, health professionals may offer the family the option to terminate the pregnancy. In these cases, it is difficult for the parents to decide whether to continue or terminate the pregnancy (Statham, Solomon, and Chitty 2000; Londras 2015). The continue or termination decision is affected by the severity of the foetal defects, the mother's personality, values, beliefs and family history, and supportive factors (Cockrill et al. 2013; Frederico et al. 2018; Sun et al. 2020). In some cultures, women may be blamed for deciding to termination or having a baby with foetal anomaly (Chaloumsuk 2013). Also ending the foetus' life is a great circumstance for women (Hassan 2015). According to Zhang et al. (2023) following foetal anomaly diagnosis, coping strategies may regulate emotions and wellbeing and emotional support is one of the coping strategies for women. Kamranpour, Noroozi, and Bahrami (2019) stated that women with foetal anomalies need their spouse's understanding, empathy and acceptance. It is shown that social, spousal and particularly family support given to the women play an active role in preventing future psychological problems in foetal anomalies (Kamranpour, Noroozi, and Bahrami 2019; Sun et al. 2020).

Parents have needs throughout the process of diagnosing foetal anomalies, pregnancy and bereavement, and perinatal palliative care programmes address these needs (Wool 2013). Most crucially, these programmes help parents to gain a sense of meaning and recognition of parenthood for their unborn child (Cote‐Arsenault and Denney‐Koelsch 2011). Appropriate clinical care by trained health professionals can mitigate the psychological impact of such incidents and possibly prevent further deterioration of parental trauma (Erlandsson et al. 2011; Lee 2012; Pullen, Golden, and Cacciatore 2012). Ongoing support for the couple must be a sustained process, starting at diagnosis and continuing throughout the delivery and postpartum period, even after the parents have transitioned home (Pullen, Golden, and Cacciatore 2012). Thus, health professionals should provide psychosocial support not only to the woman and her family who decide to terminate the pregnancy, but also to parents receiving the diagnoses of foetal anomaly or parents who want to continue a pregnancy despite the recommendation for medical termination (Mecdi Kaydirak and Aslan 2021).

The literature contains studies examining the experiences, psychosocial status and grief of parents who have experienced medical termination due to foetal anomaly. The experience has been reported as the most difficult and painful of life experiences for the mother and her spouse (Sun et al. 2020; Mecdi Kaydirak and Aslan 2021). However, the literature appears to lack research examining the relationship between social support and the anxiety, stress and depression of pregnant women diagnosed with foetal anomaly.

Since the literature states that, women need their spouse's support during foetal anomaly experience (Kamranpour, Noroozi, and Bahrami, 2019).

## 3 The Study

### 3.1 Aim

This study aimed to examine the relationship between social support and the anxiety, stress and depression of pregnant women diagnosed with foetal anomaly.

### 3.2 Research Questions

What is the mean score of the pregnant women in MSPSS and its sub‐dimensions?

What is the mean DASS—42 sub‐dimension score of pregnant women?

Are perceived social support and depression, anxiety, stress levels correlated in pregnant women diagnosed with foetal anomaly?

## 4 Methods

### 4.1 Design

This descriptive and correlational study was carried out between December 2021 and May 2022 to examine the correlation between the perceived social support and depression, anxiety and stress levels of pregnant women diagnosed with foetal anomaly.

### 4.2 Participants

The research population consisted of pregnant women presenting to the advanced prenatal evaluation unit of a university hospital. The research sample calculation was made according to the effect width (*w* = 0.3) and the one‐tailed hypothesis method, considering the number of pregnant women who applied to the clinic in 1 year (*N* = 500). G‐Power analysis was performed with *α* = 0.05 and *β* = 0.20 values at 1° of freedom according to effect widths (Kalaycıoglu and Akhanlı 2020), and the minimum number of pregnant women to be included in the sample was calculated as 126. Finally, 131 pregnant women who met the inclusion criteria were included in the sample using a simple random sampling method.

### 4.3 Inclusion Criteria

Over 18 years old,

Diagnosis of foetal anomaly arising from pregnancy check‐up,

At least 1 week of elapsed time since the diagnosis of foetal anomaly,

Ability to communicate normally,

Willingness to participate in the research.

### 4.4 Exclusion Criteria

Decision to terminate the pregnancy due to foetal anomaly.

### 4.5 Data Collection

Research data was collected face‐to‐face with the researcher in a quiet and calm environment between December 2021 and May 2022. Each data collection session took approximately 10–15 min. Data was collected using the personal information form, DASS‐42 and MSPSS.

#### 4.5.1 Personal Information Form

It consists of 26 questions prepared by researchers based on examples found in the literature (Kamranpour, Noroozi, and Bahrami 2019; Sun et al. 2020). The questions evaluate sociodemographic characteristics, general health and obstetric history.

#### 4.5.2 DASS‐42

A 3‐factor scale assessing stress, anxiety and depression and consisting of 42 questions. This is a 4‐point Likert‐type scale; high scores in each of the dimensions indicate a related problem. The total scores of the scale vary between 0 and 42 for each sub‐dimension (Bilgel and Bayram 2010). Permission to use the scale was obtained via e‐mail from the authors who adapted it into Turkish. The Cronbach alpha internal consistency coefficients were 0.89 for the whole scale, and 0.90, 0.92 and 0.92 for the depression, anxiety and stress sub‐dimensions. In our study, total Cronbach's alpha coefficient for the scale was found as 0.95.

#### 4.5.3 MSPSS

A 7‐point Likert‐type scale consisting of three sub‐dimensions and 12 items evaluating social support received from the environment. The lowest score that can be obtained from the entire scale is 12 and the highest score is 84. High scores indicate high perceived social support, while low scores indicate support is not perceived or is absent (Eker, Arkar, and ve Yaldız 2001). Permission to use the scale was obtained from the authors. Total Cronbach's alpha coefficient for the scale was 0.89, sub‐dimension Cronbach's alpha coefficient was 0.85 for family, 0.88 for friend, 0.92 for significant other. In our study, total Cronbach's alpha coefficient for the scale was found as 0.87.

### 4.6 Data Analysis

Data were analysed using the Statistical Package for Social Sciences (SPSS) v 21.0. Numbers and percentages were used for descriptive statistics. Skewness and Kurtosis values evaluated to determine normality. Independent sample T test was applied in paired groups with normal distribution. Spearman correlation test was used to assess correlation between the scales. Cronbach's alpha coefficient was calculated for the scales used and *p* < 0.05 was accepted as.

### 4.7 Ethical Considerations

Ethics committee approval for the study was obtained from Istinye University Human Research Ethics Committee (Protocol No: 21‐105, dated 29.11.2021). Departmental permission was obtained from the university hospital clinic where the research was conducted. The research was conducted in accordance with research and publication ethics specified in the Declaration of Helsinki. The purpose of the study was explained to each pregnant candidate, and informed consent was obtained.

## 5 Results

The mean age of the pregnant women was 31.10 ± 5.18, the duration of marriage was 5.93 ± 4.46 years, and the gestational week was 23.17 ± 4.44 weeks. Most of the pregnant women (78.6%, *n* = 103) were in the second trimester at the time of the study. Only five pregnant women (3.8%) stated that they did not regularly attend their pregnancy check‐ups. For 55.0% (*n* = 72) of the pregnant women, a health professional had recommended pregnancy termination, but the family had decided to continue the pregnancy. Table 1 shows the findings regarding the sociodemographic and obstetric characteristics of pregnant women (Table 1).

**TABLE 1:** Sociodemographic characteristics of pregnant women (*N* = 131).

	Min	Max	Mean ± SD
Age	20	51	31.10 ± 5.18
Duration of marriage (years)	1	25	5.93 ± 4.46
Gestation week	13	36	23.18 ± 4.44
Number of pregnancies	0	7	1.98 ± 1.24
Features		N	%
Educational status	Primary school	13	9.9
	Middle School	17	13.0
	High school	37	28.2
	Bachelor and above	64	48.9
Income status	Less than expense	16	12.2
	Equal to expense	92	70.2
	More than expense	23	17.6
Family type	Nuclear family	122	93.1
	Extended family	9	6.9
Employment status	Working	62	47.3
	Not working	69	52.7
Smoking	Yes	10	7.6
	No	121	92.4
Alcohol use	Yes	4	3.1
	No	127	96.9
Continuous drug use	Yes	29	22.1
	No	102	77.9
Chronic disease	Yes	34	26.0
	No	97	74.0

The total mean score of the pregnant women in MSPSS was 65.4 ± 14.57, and the sub‐dimension mean score was 20.70 ± 6.55 for family support, 21.43 ± 5.73 for friend support and 23.80 ± 3.85 for significant other support. The mean score of the pregnant women on DASS‐42 was 9.55 ± 8.67 for depression, 8.48 ± 8.45 for anxiety and 13.83 ± 9.48 for stress (Table 2).

**TABLE 2:** Findings on Scale total scores and rates of pregnant women (*N* = 131).

Scales	Min		Max		Mean ± SD	
MSPSS total	25		84		65.95 ± 14.58	
Family support	4		28		20.70 ± 6.55	
Friend support	8		28		21.44 ± 5.73	
Dedicated human support	8		28		23.81 ± 3.85	

DASS – 42	Min		Max		Mean ± SD	
Depression	0		38		9.56 ± 8.68	
Stress	0		50		13.84 ± 9.48	
Anxiety	0		36		8.48 ± 8.45	

DASS‐42 Scores by Level						
	Depression		Anxiety		Stress	
	*n*	%	*N*	%	*n*	%
Normal	78	59.5	76	58.0	82	62.6
Light	20	15.3	13	9.9	16	12.2
Middle	17	13.0	23	17.6	18	13.7
Forward	9	6.9	2	1.5	8	6.1
Too advanced	7	5.3	17	13.0	7	5.3

The results regarding the comparison of DASS‐42 and MSPSS were given in Table 3. There was no statistically significant difference between the depression subscale of pregnant women and the total and all sub‐dimensions of MSPSS (*p* > 0.05). A statistical difference was found between the anxiety subscale of pregnant women and the MSPSS total and friend support subscales, and that the difference occurred between the normal and moderate groups. It was determined that there was a statistical difference between the stress subscale of pregnant women and the family support and friend support subscales, and that the difference occurred between the normal and advanced groups (Table 3).

**TABLE 3:** Comparison of MSPSS scores of pregnant women according to DASS‐42 scores (*N* = 131).

DASS‐42 scores by level	MSPSS total MR	Test value	*p*	Family support	Test value	*p*	Friend support	Test değeri	*p*	Dedicated human support	Test value	*p*
Depression												
Normal	72.22	5932*	0.204	71.42	4722*	0.317	72.68	6632*	0.157	70.76	5660*	0.226
Light	54.43			58.98			54.40			48.93		
Middle	57.82			54.65			57.24			62.03		
Forward	52.33			54.33			51.22			69.44		
Too advanced	67.21			68.21			65.00			67.00		
Anxiety												
Normal^a^	72.68	***10*.*440*** * ***a > c***	0.034**	71.59	8289*	0.082	75.05^a^	***15*.*602*** * ***a > c***	0.004**	67.61	4649*	0.325
Light^b^	58.54			59.27			56.23^b^			58.08		
Middle^c^	44.61			47.02			40.83^c^			55.93		
Forward^d^	72.75			76.00			58.25^d^			96.50		
Too advanced^e^	70.00			70.65			67.97^e^			74.91		
Stress												
Normal^a^	71.85	9160*	0.054	72.49^a^	***10*.*259*** * ***a > e***	0.036**	72.04^a^	***9845*** * ***a > e***	0.043**	67.41	2488*	0.647
Light^b^	46.75			45.03^b^			45.97^b^			61.41		
Middle^c^	62.47			62.89^c^			61.14^c^			63.14		
Forward^d^	72.94			69.06^d^			74.81^d^			79.50		
Too advanced^e^	42.64			42.43^e^			43.43^e^			51.86		

Abbreviation: MR: mean rank. a: Normal anxiety score, b: Light anxiety score, c: Middle anxiety score, d: Forward anxiety score, e: Too advanced anxiety score.

* :Kruskal–Wallis test; **: *p* < 0.05 significance value.

Table 4 presents the relationship between the perceived social support level of pregnant women and DASS‐42. There is a weak negative correlation between family support, friend support and total MSPSS perceived by pregnant women and their level of depression. It was determined that there was a weak negative correlation between friend support and total MSPSS and anxiety level, and a weak negative correlation between family support and stress level (Table 4).

**TABLE 4:** Findings regarding the DASS‐42 relationship of perceived support level (*N* = 131).

	DASS‐ 42		
Scales and subscales	Depression (rs)	Anxiety (rs)	Stress (rs)
Family support	−0.231	−0.134	−0.182
	***0*.*008*** **	0.127	***0*.*038*** *
Friend support	–0.218	−0.235	−0.167
	***0*.*012*** *	***0*.*007*** *	0.056
Dedicated human support	−0.162	−0.027	−0.062
	0.064	0.762	0.483
MSPSS total	−0.236	−0.175	−0.171
	***0*.*007*** **	***0*.*046*** *	0.051

Abbreviation: rs: Spearman correlation coefficent.

** *p* < 0.01 level (two‐tailed).

* *p* < 0.05 level (two‐tailed).

## 6 Discussion

This study aimed to examine the relationship between perceived social support and depression, anxiety and stress in pregnant women diagnosed with foetal anomaly. Half the research sample (55.0%, *n* = 72) were pregnant women who had been advised to medically terminate their pregnancy due to foetal anomaly. The literature contains limited information on research variables (Skotko 2005; Kleinveld et al. 2006; Herrera et al. 2022; Buskmiller et al. 2023). For this reason, the results of research examining medical termination of pregnancy were discussed and social blogs were used to interpret the data. We anticipate that the results of this approach will contribute a different perspective to the literature.

Although their character structures and life experiences are different, the spiritual journeys of pregnant women diagnosed with foetal anomaly may resemble one another. It has been determined that the risk of depression during this difficult period is twice as high as the general population (Buskmiller et al. 2023). Among pregnant women diagnosed with foetal anomaly, it was found that women whose pregnancies were terminated had a higher risk of depression compared to women whose pregnancies continued (Herrera et al. 2022). Studies report that mothers found to be high‐risk as a result of screening tests have increased anxiety levels (Marteau et al. 1992; Kızoglu and Beydag 2022). Many expectant mothers stated that the most difficult moment in the process of diagnosis and treatment is the moment the diagnosis is first learned. After accepting the diagnosis, they stated they believed the birth process would progress positively with emotional maturity. The planned pregnancy of the mother or the level of foetal anomaly can also influence the level of anxiety experienced (Basgul 2013). The anxiety levels of women with positive screening tests from forum sites on the internet were high, characterised by statements such as, ‘I wish I hadn't taken the test’, ‘I don't know how I will spend my remaining weeks’ and ‘I don't know what to do’ (Bebek Saglıgı ve Hastalıkları 2022; Kadınlar Kulubu 2022). These emotional transitions are a psychologically normal process. In one study, pregnant women stated that they felt as if time had stopped upon initial diagnosis and they had collapsed in the face of the result (Skotko 2005). Loss is not just the physical death of a person. Should an event that is wished or expected not occur, individuals may experience feelings of loss (Bowlby 2008). For a mother dreaming of a healthy pregnancy and baby the diagnosis of foetal anomaly is a loss (Mecdi Kaydirak and Aslan 2021). This study selected participants who had known their diagnosis for at least a week; during this time, they may have experienced certain stages of acceptance and their anxiety levels therefore may have been lower. Anxiety is the feeling of worry and fear with the expectation of danger that may arise from inside or outside (Budak 2005). The fact that the foetal anomaly diagnosis of the pregnant women in the study group was clear and the results were discussed in detail by health professionals may explain their lower anxiety levels.

Stress is a situation that a person is not ready for and that creates a change in one's life and creates a crisis. The feelings of fearfulness, crying, sadness and stress experienced by women differ individually. One study determined that pregnant women who experienced loss use social support and an approach helping them to accept the ongoing situation as a way of coping with stress (Kaydırak 2018). Kleinveld et al. (2006) determined that there was no change in the anxiety level of pregnant women diagnosed with foetal anomaly and they did not have any concerns about the foetus. A study on pregnancy loss found that mothers who lost their babies during pregnancy had high levels of post‐traumatic stress disorder (Keten et al. 2015). It is known that social support mechanisms have a positively affect mental health, protect against the negative effects of stress and strengthen coping skills. This support becomes much more important for the pregnancy period. One study determined that the support received from people who are important in a woman's life has a positive effect on the pregnancy process (TAŞKIN, 2019).

A diagnosis of foetal anomaly marks the loss of a healthy baby for a couple. It is known that the support from the spouse, family and environment has a great effect on recovery from such losses. One study determined that the majority of mothers who experienced pregnancy loss received support from their spouse, family and friends during this period (Sutan et al. 2010). It has been determined that women need social support more from their partners during this period (Dekkers et al. 2019). Due to religious beliefs and social and cultural factors, the mother may not see the foetal anomaly as a loss and may accept the baby (Oner 2017). In addition, it is thought that in cases of structural anomalies compatible with life (such as cleft palate, cleft lip, prodactyly), the inability of pregnant women to see the postpartum process very clearly and not to share this situation with their relatives may cause that the pregnant women will be lack of adequate support; and the uncertainty of the postpartum newborn process may cause anxiety in pregnant women. The researchers have found a study in the literature examining the relationship between the perceived social support and depression, anxiety and stress in pregnant women diagnosed with foetal anomaly. This research determined that stress decreased as family support, friend support and multidimensional social support increased. It has been reported that various mental and psychological problems (depression, anxiety, stress, fear, etc.) may occur in pregnant women in the event of insufficient perceived social support (Bedaso et al. 2021). In a study conducted in Iran, women diagnosed with foetal anomaly reported receiving support from family, friends and others as a coping strategy. (Irani et al. 2019). The systematic review and meta‐analysis of the relationship between the perception of social support and mental health of pregnant women reported that low social support was associated with anxiety, stress and depression, but maternal and foetal conditions such as pregnancy status and the health of the foetus and such sociodemographic variables such as age and economic status could be also affected (Bedaso et al. 2021). Mecdi's study reported that women who experienced feelings of loss after the medical termination of pregnancy were negatively affected by perceived social support, the quality of the support and the many expressions people used for support (Mecdi Kaydirak and Aslan 2021). This finding explains the minimum effect of the support obtained as a result of the research on stress.

## 7 Conclusion

Most of the pregnant women diagnosed with foetal anomaly have normal depression, stress and anxiety levels. Perceived multidimensional social support is good, and there is a weak negative correlation between perceived social support and stress levels. The support received from family and friends was related to stress level, but the effect rate was low. Professional support is indicated for pregnant women diagnosed with foetal anomaly. Not only must support be provided to the pregnant woman and the people around her, but depression, stress and anxiety levels and social support mechanisms must also be evaluated.

## Ethics Statement

Ethics committee approval for the study was obtained from Istinye University Human Research Ethics Committee (Protocol No: 21‐105, dated 29.11.2021). Departmental permission was obtained from the university hospital clinic where the research was conducted. The research was conducted in accordance with research and publication ethics specified in the Declaration of Helsinki. The purpose of the study was explained to each pregnant candidate, and informed consent was obtained.

## Consent

Informed consent was obtained from all individual participants included in the study. The authors affirm that human research participants provided informed consent for publication of the manuscript.

## Conflicts of Interest

The authors declare no conflicts of interest.

## Peer Review

The peer review history for this article is available at https://www.webofscience.com/api/gateway/wos/peer‐review/10.1111/jan.16587.

## Data Availability

The data that support the findings of this study are available on request from the corresponding author. The data are not publicly available due to privacy or ethical restrictions. ÇEVİR.
